# Effects of cognitive interventions with video games on cognition in healthy elderly people: a systematic review

**DOI:** 10.1055/s-0043-1764413

**Published:** 2023-05-31

**Authors:** Graciela Akina Ishibashi, Gabriela dos Santos, Ana Paula Bagli Moreira, Cássia Elisa Rossetto Verga, Guilherme Alves da Silva, Tiago Nascimento Ordonez, Luiz Carlos de Moraes, Patrícia Prata Lessa, Sonia Maria Dozzi Brucki, Thais Bento Lima da Silva

**Affiliations:** 1 Universidade de São Paulo, Escola de Artes, Ciências e Humanidades, Departamento de Gerontologia, São Paulo SP, Brazil.; 2 Instituto Supera de Educação, São José dos Campos SP, Brazil.; 3 Universidade de São Paulo, Escola de Artes Ciências e Humanidades, Grupo de Estudos de Treinamento Cognitivo, São Paulo SP, Brazil.; 4 Universidade de São Paulo, Faculdade de Medicina, Hospital das Clínicas, Grupo de Neurologia Cognitiva e Comportamental, São Paulo SP, Brazil.; 5 Universidade de São Paulo, Faculdade de Medicina, Departamento de Neurologia, São Paulo SP, Brazil.

**Keywords:** Elderly, Cognitive Aging, Video Games, Cognition, Idoso, Envelhecimento Cognitivo, Video Games, Cognição

## Abstract

**Background** Currently, studies using video games as an intervention to improve cognitive functions in the elderly are on the rise.

**Objective** To investigate and evaluate the effects of cognitive interventions using video games on cognition in healthy elderly people published in the last ten years.

**Methods** A systematic review involving a qualitative analysis carried out between July and September 2021on the SciELO, LILACS and MEDLINE databases..

**Results** A total of 262 articles were identified in the initial search. After exclusion of duplicates, analysis of titles/abstracts and of the full text, a final total of 9 studies were included in the review. The objectives of the studies included investigating the effects on cognition of cognitive training (CT) programs using video games compared to programs using entertainment games or to low-intensity CT games. Despite the growing number of studies, many of them were focused on cognitive rehabilitation in elderly people with some degree of cognitive impairment, and few involved training among healthy elderly people.

**Conclusion** According to the studies analyzed, the interventions involving CT with video games promoted significant improvements in processing speed and working memory, but no improvements in executive functions.

## **Resumo**

**Antecedentes** Atualmente, estão em alta estudos que utilizam *video games* para melhorar as funções cognitivas em idosos.

**Objetivo** Investigar e avaliar os efeitos de intervenções cognitivas com *video games* na cognição de idosos saudáveis publicadas nos últimos dez anos.

**Métodos** Estudo de revisão sistemática com análise qualitativa realizado entre julho e setembro de 2021, das bases de dados SciELO, LILACS e MEDLINE.

**Resultados** Foram identificados 262 estudos na busca inicial. Após a exclusão de estudos duplicados, análise dos títulos e resumos, e análise integral dos estudos, nove estudos foram incluídos na revisão. Os objetivos de alguns estudos incluíam investigar os efeitos na cognição de programas de treino cognitivo (TC) com *video games* comparados aos de programas com jogos de entretenimento ou com jogos com baixa intensidade de TC. Apesar do crescente número de estudos, muitos centravam-se na reabilitação cognitiva em idosos com algum comprometimento cognitivo e poucos realizaram o treino em idosos saudáveis.

**Conclusão** Pelos estudos analisados, as intervenções de TC com *video games* apresentaram melhorias significativas na velocidade de processamento (VP) e na memória operacional (MO); por outro lado, não foram observadas melhorias nas funções executivas.

## INTRODUCTION

The number of studies investigating cognitive aging has grown in recent years, given that cognitive performance declines as part of the normal aging process, with slower processing speed (PS) and reduced performance of the working memory (WM). These impairments are often of particular concern among older adults and their family members because of the risk of progression to dementia, as in Alzheimer disease. The literature 1 shows that the quality of execution of complex activities of everyday living can change as part of the normal process of healthy aging.

Cognitive interventions, more specifically cognitive training (CT), can serve as a method of both prevention and rehabilitation of some cognitive deficits. Technological advances enable CT through the use of video games, an approach that renders tasks more engaging and fun, increasing participant motivation and reducing abandonment rates, 2 with the added advantage of being more accessible to the general public. 3

In this context, cognitive decline is correlated with cognitive reserve. Greater cognitive reserve is associated with the prevention of dementia, but in order for the interventions to yield benefits, the activities must be performed over a relatively long period without loss of motivation. This interest can be achieved through the use of cognitive computer games, leisure-time activities, social interaction, intellectual stimulation etc. Social interactions and intellectual stimulation have been shown to reduce the risk of dementia. 4

Video games can be divided into CT games and entertainment games. 5 While CT games are designed to train cognitive functions and are developed by neuroscientists, games for entertainment are focused on individual leisure and fun, such as the game Super Mario Bros.

For the present review, we will use serious games that have the goal of not only entertaining, but also of teaching and training cognitive functions such as memory, attention etc., which differs from computerized CT, which focuses only on training cognition, regardless of the participant's enjoyment. This type of game has been used as a way of promoting cognitive stimulation, for example, in older adults during the period of social isolation caused by the coronavirus disease 2019 (COVID-19) pandemic. 6

The study by Belchior et al. 7 involved the use of video games and brain training. Video games are essentially designed to entertain the general public. By contrast, brain-training games are especially designed to train an ability in a specific population. A study 8 based on a video game intervention in older adults reported improvements in WM and sustained attention relative to the control group. Similar results were found in the study by Toril et al., 9 in which a video game intervention enhanced visuospatial working memory, episodic memory, and short-term memory in healthy older adults.

Ordonez et al. 10 conducted a study using a cognitive stimulation program on a Japanese video game equipment adapted for use by Brazilian older adults. The results revealed improvements in cognitive performance, specifically on the memory and language domains, and there was a reduction in the level of anxiety and in the rate of memory complaints.

The study by Assis 11 used video games controlled by body movements in the neurorehabilitation of older adults with mild cognitive impairment (MCI) at three levels of schooling (primary, secondary, and higher education). The results showed improvements in the memory of participants with primary and secondary level of schooling, but no improvement in the group with higher education.

Also in this context, a systematic review and meta-analysis 12 evaluated the effects of video games, in relation to the control condition (no game), on cognitive functions in the elderly. The authors 12 obtained 27 studies that met the inclusion criteria, and they concluded that video game interventions improved the memory of the participants; however, they found no improvement in other cognitive functions.

Finally, another meta-analysis 13 aimed to summarize the current level of evidence for brain training using commercial computerized cognitive games (ccCGs) in healthy older adults. It has been documented 3 5 9 that commercially available cognitive games are effective in improving PS, WM, executive functions, and verbal memory.

However, despite the existence of several studies 12 that used electronic games as an intervention to improve cognitive functions in the elderly, specific games for healthy elderly people are hardly addressed to this contingent. With the rapid advancement of technologies and the recent COVID-19 pandemic, the population started to make more use of social networks, video games, and online news, for example. Thus, the aim of the present study was to investigate and evaluate the effects of cognitive interventions with video games on the cognition of healthy elderly people in studies published in the last ten years.

## METHODS

A systematic review involving a qualitative analysis was conducted. All relevant scientific articles published in Portuguese, English or Spanish were retrieved from the SciELO, LILACS, and PubMed electronic databases. From the data obtained on PubMed, we noticed a large gradual and accelerated increase in studies with video games in the last 10 years; therefore, in the present review, we have searched for articles published between 2011 and 2021 (until the beginning of the article's completion).

Using the Population, Phenomena of Interest, and Context (PICo) method, the following research question was defined: what are the effects of interventions with video games, in studies published in the last ten years, on the cognition of healthy older people? The population is healthy older people, the phenomena of interest, effects on cognition, and the context, cognitive interventions with video games published in the last ten years. After this process, the eligible descriptors were selected in the Descriptors in Health Sciences (Descritores em Ciências da Saúde, DeCS, in Portuguese) system to serve as keywords for the search for articles published in Portuguese, English and Spanish on the SciELO, LILACS and PUBMED databases. The following search terms were used: idoso* OR *elder* OR older person* OR *older people* OR senior citizen* OR *elderly* OR *aging people* OR aging person* OR older adult* OR anciano* OR persona* mayor* and intervenção* cognitiva* OR cognitive intervention* OR *treino cognitivo* OR *cognitive training* OR *treino de memória* OR *memory training* OR intervención* cognitiva OR *entrenamiento cognitivo* OR *entrenamiento de memoria* and jogo* OR *juego* OR *game* OR *online* OR *on-line* OR *computerized* OR *video game* OR *juego* OR *en línea* OR *computerizado* .

The inclusion criteria were studies with interventions involving serious games; healthy participants aged ≥ 60 years; articles published in scientific journals since 2011; and use of cognitive and/or neuropsychological tests to determine the effects of the interventions. The exclusion criteria were: publications of Masters dissertations, book chapters, doctoral theses, letters to the editor, case studies, systematic reviews, meta-analyses, and research protocols; participants aged < 60 years or presenting cognitive impairment; studies performed in residential care homes, such as long-term care facilities (LTCFs); studies failing to report the effects of the intervention on the cognitive performance of the participants; and studies combining multimodal interventions (such as physical training, action games, and nutritional components).

The Preferred Reporting Items for Systematic Reviews and Meta-Analyses (PRISMA) 14 statement was used for the identification, screening, and eligibility stages of the studies, and two reviewers acted independently throughout the stages. The initial identification of studies entailed searches of the aforementioned databases. During the screening stage, duplicate studies were removed, and titles and abstracts were analyzed by applying the inclusion and exclusion criteria. Differing opinions were resolved through a conference with all reviewers. For the eligibility stage, the selected studies were read in full and analyzed according to the same criteria. The studies remaining after this stage were included in the review. Only peer-reviewed articles were considered for the systematic review.

The scope of the systematic review was registered on the International Prospective Register of Systematic Reviews under number CRD42021267903 (Ishibashi et al., 2021). In addition, the included studies were rated for quality according to the Downs and Black Checklist. 15 This evaluation tool devised by Downs and Black comprises 27 items distributed among 5 subscales: reporting or assessment (10 items), external validity (3 items), internal validity of the measurements described and outcome bias (7 items), confounding factors (6 items) and power (1 item). The checklist items were assigned scores of 0 or 1, except for the item assessing the reporting of confounding factors, whose scores ranged from 0 to 2 points, and the item assessing the reporting of the study power (item 27), modified as per other studies, 16 17 18 whose original scores from 0 to 5 points were changed to scores of 0 or 1 point, with 1 meaning that the article reported calculation of the power and/or sample size and 0, that these calculations were not performed. Thus, the total scores on the checklist ranged from 0 to 28 points. In order to improve the reading of the data obtained, the scores were converted into percentages for each domain, and an overall mean total score for all domains was calculated. A system to classify the quality of the articles was defined: ≤ 0.39–poor; 0.40 to 0.69–regular; 0.70 to 0.79–good; and ≥ 0.80–excellent.

## RESULTS

**Figure 1:**
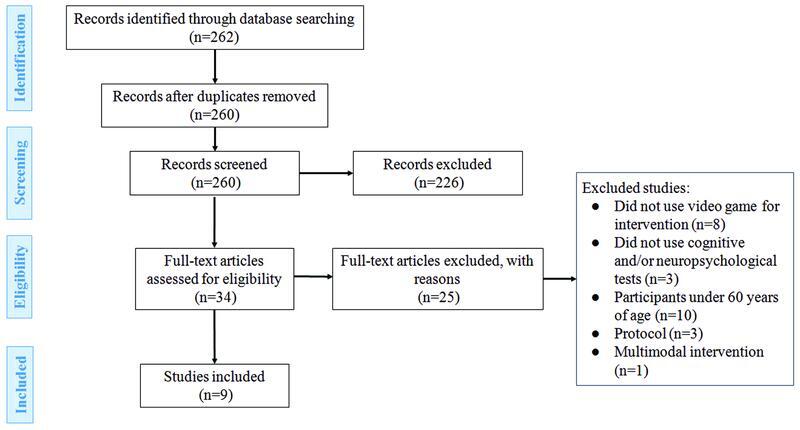
Flow diagram of the selection process of the present study.

A total of 262 studies were retrieved in the initial search, 2 of which were subsequently excluded because they were duplicates. Titles and abstracts of the remaining 260 studies were read, and then, screened for selection according to the inclusion and exclusion criteria. Thus, 34 articles were read in full and, after an in-depth analysis, a total of 9 studies were included in the review. The screening and selection process is depicted in the PRISMA flow diagram ( Figure 1 ).

Nine studies involved interventions with CT games. 4 19 20 21 22 23 24 25 26 The sample size ranged from 31 to 681 subjects, with ages ranging from 60 to 85 years. The length of the intervention ranged from 5 to 12 months, with a minimum of 8 and a maximum of 720 sessions lasting from 30 to 120 minutes each. Follow-up assessments took place within one year of the interventions ( Table 1 ).

**Table 1:** Studies involving interventions with CT games

Authors (year)	Participants	Intervention			Cognitive and/or neuropsychological tests	Results
		Experimental group	Control group	Frequency and duration		
Bozoki et al. 19 (2013)	n = 60; age: 60–80 years.	n = 32; Photoflaw (visual attention, visual WM), Headline Clues (verbal memory, reasoning and PS), Sokoban (spatial executive processing and non-verbal reasoning) and Keep It In Mind (WM).	n = 28; Thoughts In Motion (online video news clips of the day), Sound Thinking (audio news clips of the day), and Headline Clues (textual news stories of the day, with pictures).	6 weeks; 5 sessions of at least 30 minutes per week; follow-up: after 2 months.	MON, IDN, CPAL, CPAR, OCL, OWL, OWLR, ONB, and PRD.	Learning effects for the suite of games were relatively small due to large variations in amount of time devoted to game play, and reluctance to pursue more challenging levels, in which participants had full independence in terms of choices during the intervention.
Lee et al. 20 (2013)	n = 31; age: 60–70 years.	n = 15; brain-computer interface with a card-pairing memory game.	n = 16; passive control.	8 weeks; 3 sessions of 30 minutes per week.	RBANS	The memory training game with neurofeedback showed promise in improving memory and attention in healthy elderly people. The adherence rate was very high, suggesting a high level of motivation among participants.
Simon et al. 21 (2018)	n = 82; mean age: 73.1 (±6.1) years.	n = 41; adaptative Cogmed program.	n = 41; non-adaptative Cogmed program.	5 weeks; 5 sessions of 40 minutes per week.	VMST, DST, WMS-R, TMT-A and B, COWAT, and Semantic Fluency.	The WM training game evidenced improvement in trained tasks and on an untrained task dependent upon WM and PS. Gains were linked to continuously challenging level of task difficulty.
Yeo et al. 22 (2018)	n = 227; Age: 60–80 years.	n = 109; BRAINMEM (attention and WM).	n = 118; passive control.	8 weeks; 3 sessions of 40 minutes per week.	RBANS and RBMT-II.	The game intervention promoted no general gains or improvements in cognitive performance or in specific cognitive domains, such as attention and delayed memory. Neither were there improvements in memory performance in everyday tasks.
Iizuka et al. 4 (2019)	N = 72; mean age: 76.8 (±5.4) years.	n = 25: Go game group lessons; n = 25: Go game individual lessons.	n = 22; lecture on health maintenance.	12 weeks; 1 session of 60 minutes per week; follow-up: after 3 months.	VMST, DST, LM, TMT-A, and TMT-B.	The study results showed the intervention could improve visual WM in older adults. These findings suggest increased social interaction by playing board games face to face is more effective to improve cognitive function than playing alone.
Perrot et al. 23 (2019)	n = 36; age: 60–71 years.	n = 12; Kawashima Brain Training.	n = 12:Super Mario Bros.; n = 11: passive control.	8 weeks; 3 sessions of 60 minutes per week.	Matrix reasoning, Stroop, TMT, DSST, Corsi clock, spatial relation, and number comparison.	Both types of training promoted significant improvements in some aspects of cognitive functions of participants, such as inductive reasoning. The CT game promoted less transfer to cognitive tasks than the action video game.
Faust et al. 24 (2020)	n = 151; age: ≥ 60 years.	n = 39: InSight (games training visual processing and memory); n = 38: Brain Fitness (games training auditory processing, memory, and language); n = 37: both games combined (visual and auditory).	n = 37; passive control.	8–10 weeks; 30–40 sessions of 30–40 minutes per week (at least 900 minutes); follow-up: after 3 months.	HVLT, RBANS, and WAIS.	Visual training yielded transfer effects to WM in the main sample and transfer to PS in the pilot sample. There were no comparable transfer effects for auditory training. Combined visual and auditory training failed to yield synergistic effects or any significant transfer effects.
Lee et al. 25 (2020)	n = 59; age: 65–79 years.	n = 29; CT program with 17 games.	n = 30; 13 commercially available computer games.	10 weeks; 5 sessions of 42 minutes per week.	DSST, pattern comparison, letter comparison, N-back, visual short-term memory, spatial working memory, trail-making, attentional blink, Stroop, general life satisfaction, self-efficacy, perceived stress, odor identification, standing balance, and Flanker.	Home-based adaptive CT participants outperformed their counterparts who played non-adaptive CT games in cognitive performance. Improvements were greatest for PS and WM.
West et al. 26 (2020)	n = 69; age: ≥ 80 years.	n = 39; CogniFit Personal Coach.	n = 30; CogniFit.	8 weeks; 3 sessions of 60 minutes per week; follow-up: after 3 months.	Word List Memory, Logical Memory Story A, Target Cancellation Tests, TMT-A and TMT-B, DSST, DST, Similarities, BNT, and Category Fluency and Letter Fluency tests.	Using linear mixed models, there were no significant differences between the CT and control group on language functioning, attention, executive functioning, or memory.

Abbreviations: BNT, Boston Naming Test; COWAT, Controlled Oral Word Association; BRAINMEM, the training system has attention, working memory, and delayed recall game components; CPAL, Continuous Paired Association Learning; CPAR, Continuous Paired Association; CPQ, Computer Proficiency Questionnaire; CT, cognitive training; DSST, Digit Symbol Substitution Test; DST, Digit Span Test; DVT, Digit Vigilance Test; FSS, The Flow State Scale; HVLT, Hopkins Verbal Learning Test; IADL, Instrumental Activities of Daily Living; IDN, Identification; LM, The logical memory; MDPQ, Mobile Device Proficiency Questionnaire; MON, Monitoring; n-Back tasks, Simultaneous storage and manipulation of information; OCL, One Card Learning; ONB, One-back; OWL, One Word Learning; OWLR, One Word learning. n-Back tasks, Simultaneous storage and manipulation of information. PRD, Card Prediction; PS, processing speed; RAPM, Raven Advanced Progressive Matrices; RAVLT, Rey Auditory Verbal Learning Test; RBANS, Repeatable Battery for the Assessment of Neuropsychological Status; RBMT-II, Rivermead Behavioral Memory Test; SDMT, Symbol Digit Modalities; Stroop, Stroop Color and Word; TMT, Trail-Making Tests; TMT-A and TMT-B, parts A and B of the Trail-Making Test; UFOV, Useful Field of View; UMCFAB, University of Miami Computer-Based Functional Assessment Battery; VMST, Visual Memory Span Test; WAIS, Wechsler Adult Intelligence Scale; WM, Working memory; WMS-R, Wechsler Memory Scale-Revised; OWLR, One Word learning; N-back, tasks simultaneous storage and manipulation of information.

Lastly, according to the scores for the categories of the methodological quality check list, none of the articles scored < 0.71 points, and 9 scored > 0.78, attaining good quality rating. The mean overall score of the articles across all categories was of 0.81 out of 1.0 in terms of fulfilling the Downs and Black methodological quality requirements. The scores for each domain were: reporting – 0.97; external validity – 0.20; bias/internal validity – 0.66; confounding – 0.98; and power – 0.90 ( Table 2 ).

**Table 2:** Results of the Downs and Black Checklist for the present systematic review

Downs and Black Checklist	n	Mean	SD	Minimum	Median	Maximum
Reporting score (converted)	10	0.97	0.04	0.91	1.00	1.00
External validity score (converted)	10	0.20	0.17	0.00	0.33	0.33
Internal validity and outcome bias score (converted)	10	0.66	0.10	0.57	0.64	0.86
Confounding factors score (converted)	10	0.98	0.05	0.83	1.00	1.00
Power score (converted)	10	0.90	0.32	0.00	1.00	1.00
Overall score (converted)	10	0.81	0.04	0.71	0.82	0.86
Overall Score (original, no conversion)	10	22.70	1.16	20.00	23.00	24.00

Abbreviation: SD, standard deviation.

The games used in some studies are available on the internet: *Cogmed* ( https://www.cogmed.com/ ) and *CogniFit Personal Coach* ( https://www.cognifit.com/ ).

## DISCUSSION

The objective of the present study was to carry out a systematic review of studies investigating and assessing the effects on the cognition of healthy older adults of cognitive interventions using video games. A total of nine eligible studies were retrieved with objectives such as investigating the effects on cognition of CT programs using video games compared with entertainment game programs 23 25 or low-level CT game programs. 19 21 26 A total of four studies 4 20 22 24 performed CT using video games in the training group, but not in the control group.

Of the nine studies involving CT interventions using video games, six reported significant improvements in the cognitive performance of the participants. 4 20 21 23 24 25 The study by Lee et al. 25 showed enhanced cognitive performance, with most significant improvements for WM and PS. The findings of the study by Lee et al. 20 showed improvements in memory and attention, promoting a high participant adherence rate. The study by Iizuka et al. 4 showed improvement in visual WM and also noted that the increased social interaction by playing board games face to face is more effective to improve cognitive function than playing alone. Perrot et al. 23 found improvements in inductive reasoning and that the CT game promoted less transfer to cognitive tasks than the action video game. The study by Simon et al. 21 showed evidence of performance improvement on trained tasks and on an untrained task dependent on WM and PS.

Corroborating these results, in the study by van Muijden et al., 27 which involved a CT intervention using online games in healthy older adults, the authors showed that this type of intervention can, besides promoting gains in PS, promote the transfer of acquired skills to measures of inhibition and inductive reasoning. The results of the study by Simpson et al., 28 in which the participants performed online computer-based CT, showed that 21 days of CT can yield improvements in PS for responses to simple and complex stimuli.

Finally, of the studies that promoted significant improvements in cognitive performance, Faust et al. 24 showed that visual training yielded transfer effects to WM, but combined visual and auditory training failed to promote any significant transfer effects. Replicating the results of auditory training, Smith et al. 29 found no significant transfer effect.

The other three studies 19 22 26 included in the present review, which trained more than one cognitive ability, showed no significant improvements. For example, in the study by Bozoki et al., 19 learning effects for the suite of games were relatively small due to the great variation in the amount of time devoted to game play and reluctance to pursue more challenging levels, as participants had full independence in terms of choices during the intervention. Similarly, West et al. 26 using linear mixed models, found no significant differences found between the CT and control groups in terms of language functioning, attention, executive functioning, or memory. In the study of Yeo et al., 22 the game intervention promoted no general gains or improvements in cognitive performance or in specific cognitive domains, such as attention and delayed memory. Moreover, there were no improvements in memory performance in everyday tasks.

For studies that do not show improvements in cognition, one hypothesis would be the level of difficulty of the game and the freedom that some authors gave to the elderly to choose the difficulty and amount of time playing the game, unlike those that showed improvements. In the studies that presented superior performance, it was evidenced that the minimum duration of the intervention was o eight weeks and the sample consisted of younger elderly people.

In conclusion, the results of the present review revealed that half of the studies showed significant improvements in cognitive performance, whereas the other half failed to observe any improvement.

The feasibility issues reported in the studies reviewed included limited access to the internet at home, great variation in the amount of time devoted to game play, a reluctance to pursue more challenging levels, a sample of older adults with an active lifestyle participating in cognitive activities, and a lack of concern regarding digital inclusion and digital literacy. Participant engagement proved a barrier, and a strategy to improve engagement used by Bozoki 19 was that participants knew that if they played the game 5 times for 30 min, they would be paid US$ 5 per week. Another potential strategy would be accompanying progress using monitors to motivate the participants.

Taken together, the results of the present review showed that most interventions using video games improved WM and PS. Finally, as a limitation of the present study, we identified the scarce number of articles on healthy elderly people in the literature. Few studies used serious games as an intervention and its effects on psychosocial variables and the quality of life of healthy elderly people. Therefore, for future research, it would be interesting to address this issue.

We can consider as a limitation of the present study the fact that we may not have found, during the search stage, articles that could be part of the present review, so we consider important the performance new systematic reviews that use other search terms.
